# An observational study of intubation success rates and rescue airway techniques among 7256 pre-hospital physician intubations of trauma patients

**DOI:** 10.1186/1757-7241-22-S1-P13

**Published:** 2014-07-07

**Authors:** Kate Crewdson, David J Lockey

**Affiliations:** 1 London’s Air Ambulance, Royal London Hospital, London, United Kingdom

## Background

Effective airway management is a priority in early trauma management. Data on physician pre-hospital intubation is limited, despite its worldwide practice [1]. This study was conducted to establish intubation success rates in a physician-led system and examine the frequency and management of failed intubation in pre-hospital trauma patients. Failed intubation rates of anaesthetists and non-anaesthetists were also reviewed.

## Method

A retrospective database review was conducted to identify trauma patients undergoing pre-hospital advanced airway management between September 1991 and December 2012. Intubation success rates and success rate of individuals and by speciality were recorded. The use and success of rescue techniques were also established.

## Results

The doctor– paramedic team attended 28,939 trauma patients; 7256 (25.1%) required advanced airway management. Forty-six patients (0.6%) had immediate surgical airway performed without any attempted intubation. Of the remaining 7210 patients, intubation was successful in 7158 (99.3%). Rescue surgical airways were performed in 42 patients, seven had successful insertion of supraglottic devices, two patients had supraglottic device insertion and a surgical airway. One patient was allowed to breathe spontaneously with bag-valve-mask support during transfer. All rescue techniques were successful.

Non-anaesthetists performed 4394 intubations and failed to intubate in 41 cases (0.9%); anaesthetists performed 2587 intubations and failed in 11 (0.4%) (p= 0.02). Forty-one of 186 doctors (22%) had at least one failed intubation.

## Conclusion

This study reports the largest series of physician pre-hospital intubations. The reported success rate (99.3%) is consistent with other published series (median 99.1%) [1]. All rescue airway techniques were successful. Non-anaesthetists were twice as likely to have to perform a rescue airway intervention than anaesthetists and this difference was statistically significant. Surgical airways were performed using a standard surgical technique. The rate of surgical airway reported here (0.7%) is lower than in many other physician-led series (median 3.1%, range 0.1%-7.7%) [2,3].
